# An outer membrane vesicle‐associated two‐track model for enterotoxigenic *Bacteroides fragilis* oncogenesis

**DOI:** 10.1002/1878-0261.70357

**Published:** 2026-11-05

**Authors:** Kangchan Choi, Sang‐Hyeon Yoo, Ki‐Jong Rhee

**Affiliations:** ^1^ Regeneration Medicine Research Center Yonsei University Wonju College of Medicine Republic of Korea; ^2^ School of Medicine Trinity Medical Sciences University Kingstown Saint Vincent and the Grenadines; ^3^ Department of Biomedical Laboratory Science, College of Health Sciences, MIRAE Campus Yonsei University Wonju Republic of Korea

**Keywords:** bacterial oncogenesis, *Bacteroides fragilis* toxin (BFT), enterotoxigenic *Bacteroides fragilis* (ETBF), host‐pathogen interaction, outer membrane vesicles (OMVs)

## Abstract

Enterotoxigenic *Bacteroides fragilis* (ETBF) is a pro‐carcinogenic pathobiont thought to drive oncogenesis solely through E‐cadherin cleavage by its metalloprotease toxin, *Bacteroides fragilis* toxin (BFT). This canonical framework leaves three paradoxes unresolved: the cell type selectivity of BFT, the cytosolic engagement of NOD1 by an extracellularly secreted toxin, and the divergent epidemiology of BFT isoforms. The recent identification of claudin‐4 as the obligate BFT receptor resolves the first but sharpens the others. Here we propose an outer membrane vesicle (OMV)‐associated two‐track model. In Track 1, the surface program, claudin‐4‐dependent docking drives E‐cadherin ectodomain cleavage, junctional disruption and β‐catenin/Th17‐driven inflammation. In Track 2, the proposed intracellular program, OMV‐mediated endocytosis delivers OMV‐anchored BFT‐1 into the endocytic compartment, where endosomal escape would permit cytosolic NOD1 engagement and activation of a GAK–NUMB–NOTCH1–HEY1 axis linked to cancer stem cell expansion and chemoresistance—an axis so far demonstrated in breast cancer cells, not in colonic epithelium. We further hypothesize an intrinsic isoform bias: BFT‐2 toward surface cleavage, BFT‐1 toward intracellular NOD1 engagement. This spatially resolved model unifies recent mechanistic advances with unexplained epidemiological patterns.

## Graphical abstract

Enterotoxigenic *Bacteroides fragilis* is a gut bacterium linked to bowel cancer. We propose that its toxin acts along two routes: at the cell surface, cutting the adhesion protein E‐cadherin and triggering inflammation; and inside the cell, delivered by bacterial vesicles, switching on NOD1 and cancer stem cell programs. Different toxin variants may prefer different routes.

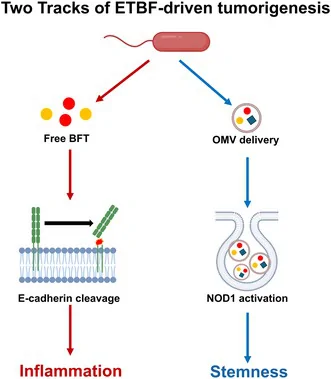

**Abbreviations**
ALDH: aldehyde dehydrogenase
BFT: *Bacteroides fragilis* toxin
CLDN4: claudin‐4
CLN4sol: soluble claudin‐4 analogue
CMS4: consensus molecular subtype 4
CRC: colorectal cancer
CSC: cancer stem cell
EC4: extracellular domain 4
EMT: epithelial‐to‐mesenchymal transition
ETBF: enterotoxigenic *Bacteroides fragilis*
GAK: cyclin G‐associated kinase
ICI: immune checkpoint inhibitor
KO: knockout
MPII: metalloproteinase II
MSI‐H: microsatellite instability high
NICD1: NOTCH1 intracellular domain
NOD1: nucleotide‐binding oligomerization domain‐containing protein 1
NTBF: non‐toxigenic *Bacteroides fragilis*
OMV: outer membrane vesicle
SEC‐MALS: size‐exclusion chromatography with multi‐angle light scattering
SP: signal peptide
Treg: regulatory T cell

## 1 Introduction

Enterotoxigenic *Bacteroides fragilis* (ETBF) is increasingly recognized as a keystone pathobiont in colorectal carcinogenesis [1]. Epidemiologic studies demonstrate a 2.54‐fold increased risk of colorectal cancer (CRC) associated with ETBF colonization [2], and murine models have firmly established its causal role in tumorigenesis [3]. The principal virulence factor of ETBF is *Bacteroides fragilis* toxin (BFT), a zinc‐dependent metalloprotease classically understood to act exclusively at the epithelial surface [4]. By cleaving the E‐cadherin ectodomain [5], BFT disrupts adherens junctions and triggers a β‐catenin and NF‐κB/STAT3‐driven inflammatory cascade [6]. This canonical surface‐cleavage model explains many critical features of ETBF‐associated tumor promotion, including IL‐17‐driven mucosal inflammation [7].

The biological relevance of ETBF extends beyond these population‐level risk associations. The *bft* locus is enriched not only in established colorectal tumors [8] but also in precancerous mucosal lesions [9], suggesting that ETBF exerts oncogenic influence at early stages of neoplastic transformation [10]. Crucially, serological data indicate that ETBF exposure can precede cancer diagnosis [11], supporting a temporally relevant causal relationship rather than simple co‐occurrence. Furthermore, experimental systems reveal that ETBF promotes mesenchymal transcriptional remodeling in CRC organoids and establishes systemic premetastatic niches in extra‐colonic tissues [12]—effects that extend well beyond what acute E‐cadherin cleavage alone would predict. Collectively, this evidence positions ETBF as a biologically active driver capable of reshaping epithelial, immune, and stromal states over a prolonged oncogenic time course [13].

Three critical unresolved paradoxes, each unexplained by surface proteolysis alone, make the limitations of this canonical model particularly consequential. First, BFT responsiveness is markedly cell type selective despite strict conservation of the E‐cadherin cleavage sequence across epithelia [14], implying the existence of undefined upstream host determinants. Second, recent findings demonstrating that BFT‐1 directly engages cytosolic NOD1 to drive cancer stem cell (CSC) expansion present a profound topological paradox for an extracellularly secreted toxin. Third, the three naturally occurring BFT isoforms (BFT‐1, BFT‐2, BFT‐3) exhibit non‐random epidemiological patterns—such as BFT‐1's dominance in adult CRC and BFT‐2's association with pediatric diarrhea [15]—that are difficult to reconcile with a single, redundant proteolytic mechanism.

The claudin‐4 discovery both clarifies the molecular basis of Track 1 and deepens the remaining two paradoxes, thereby sharpening the explanatory burden that an adequate model of ETBF carcinogenesis must bear. White et al. [16] demonstrated that claudin‐4 single knockout confers approximately 20‐fold resistance to BFT‐mediated E‐cadherin cleavage in HT29 colonic epithelial cells, rising to 500‐fold in claudin‐4/claudin‐3 double‐knockout cells; genetic rescue with GFP‐tagged claudin‐4 fully restores sensitivity. Direct binding between BFT and claudin‐4 was confirmed by co‐immunoprecipitation, pulldown assays with a soluble claudin‐4 analogue (CLN4sol), and size‐exclusion chromatography with multi‐angle light scattering (SEC‐MALS), which resolved a stable 1 : 1 stoichiometric BFT : CLN4sol complex [16]. Critically, the CRISPR screen did not recover host proteases or dedicated intracellular signaling proteins as BFT effectors; instead, the hit list was dominated by plasma membrane‐localized proteins and secretory pathway components (SEC23B, GORASP2) that localize proteins to the cell surface [16]. This definitively establishes that free, claudin‐4‐bound BFT acts exclusively at the extracellular face of the plasma membrane without crossing it—an observation fully consistent with alphafold 3 modeling of the claudin‐4 : BFT : E‐cadherin ternary complex, which positions BFT at the plasma membrane surface poised to cleave E‐cadherin residues Lys697‐Val701 through the BFT active site [16]. Two consequences for the remaining paradoxes are immediate. First, if free BFT is definitively confined to the extracellular surface, then cytosolic NOD1 engagement by BFT‐1 cannot occur through soluble toxin diffusion—it demands a compartment‐bypassing delivery mechanism. Second, because all three BFT isoforms share the same claudin‐4‐binding architecture, differential receptor affinity cannot account for their divergent disease associations; the functional asymmetry must arise downstream of receptor engagement, at the level of catalytic activity, membrane interaction, or intracellular trafficking.

In this review, we propose that both remaining paradoxes are coherently resolved by an outer membrane vesicle (OMV)‐associated two‐track model. OMVs, as encapsulated delivery vehicles capable of endocytic internalization [17], provide the topological solution to the NOD1 paradox: OMV‐mediated endocytic internalization delivers OMV‐anchored BFT‐1 to the intracellular compartment, where endosomal escape enables cytosolic NOD1 engagement without requiring free BFT to traverse the plasma membrane. This bifurcated architecture gives rise to two spatially distinct but cooperative programs. Track 1 is the surface program—initiated by claudin‐4‐dependent BFT docking at the plasma membrane, culminating in direct E‐cadherin ectodomain cleavage, β‐catenin‐linked proliferative signaling, and Th17‐driven inflammatory amplification. Track 2 is the intracellular program—supported by OMV‐mediated delivery, defined by BFT‐1‐dependent NOD1 stabilization, GAK–NUMB–NOTCH1–HEY1 axis activation, and CSC expansion with associated chemoresistance. We further hypothesize that BFT isoforms possess intrinsic structural biases toward these specific programs—with BFT‐2 structurally optimized for claudin‐4‐mediated surface cleavage (Track 1) and BFT‐1 equipped for intracellular NOD1 engagement (Track 2)—providing a mechanistic basis for the non‐random epidemiological distributions that have long resisted explanation. This integrated framework bridges the gap between claudin‐4‐dependent junctional injury at the cell surface and chronic, stemness‐driven oncogenic transformation driven from within the cell (Fig. 1).

**Fig. 1:**
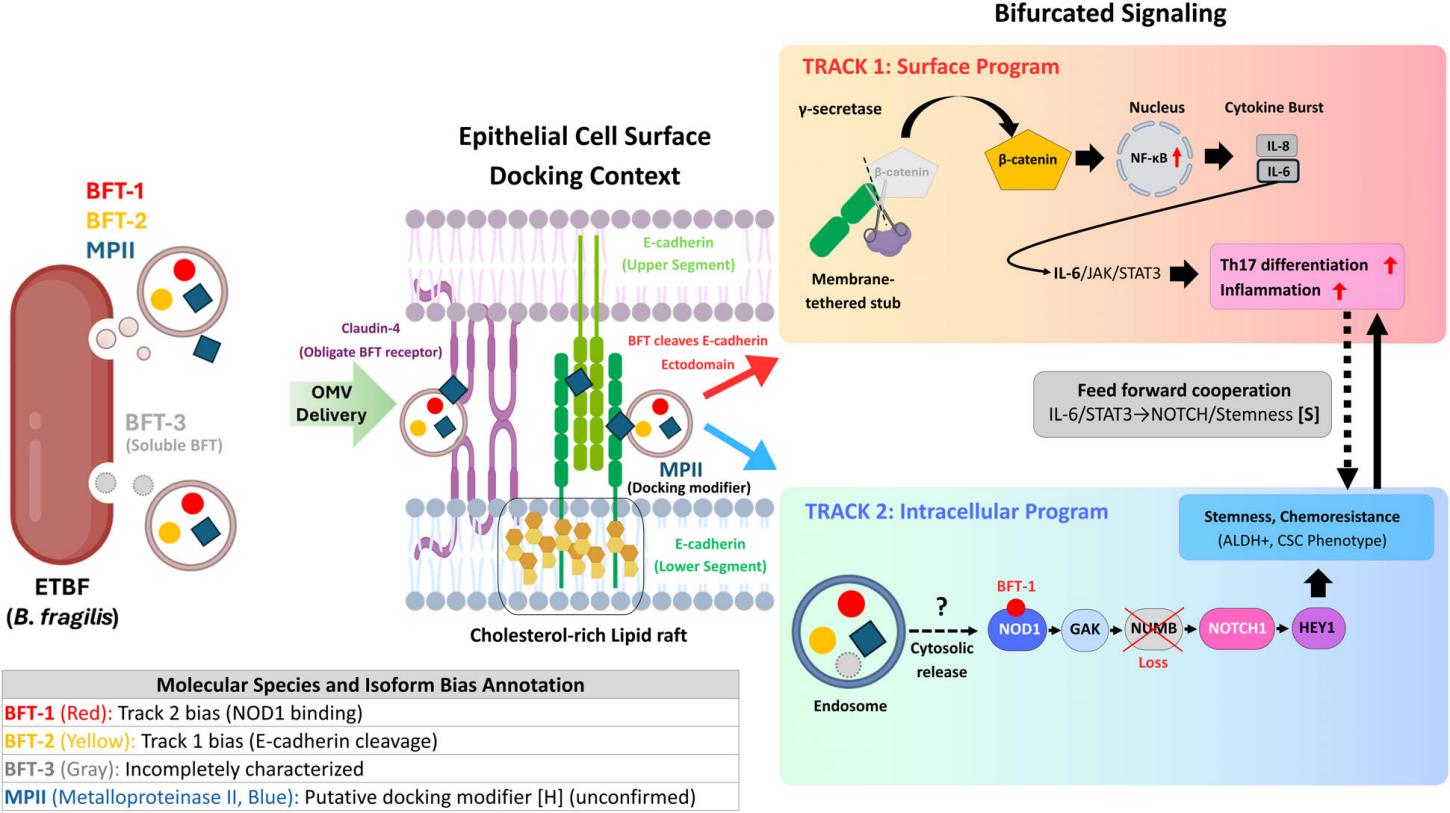
OMV‐associated two‐track model of ETBF oncogenesis. ETBF‐derived outer membrane vesicles (OMVs) carry BFT isoforms and engage the colonic epithelial surface through a claudin‐4‐dependent receptor interaction (established [E]; White et al., 2026) that recruits BFT to the plasma membrane for direct E‐cadherin ectodomain cleavage. The permissive docking context is further shaped by E‐cadherin‐rich junctional architecture and cholesterol‐sensitive membrane organization; a candidate co‐modulatory role for metalloproteinase II (MPII), the second metalloprotease encoded at the B. fragilis pathogenicity island, is depicted as a putative docking modifier [H]; the principal supporting study has received an Expression of Concern, and this interaction is not confirmed. Signaling then bifurcates into two spatially distinct programs. Track 1 (surface program): Claudin‐4‐dependent BFT docking at the plasma membrane enables direct E‐cadherin ectodomain cleavage; the membrane‐tethered stub undergoes γ‐secretase processing; β‐catenin nuclear translocation drives proliferative programs; NF‐κB/STAT3/Th17‐associated inflammation is amplified. Track 2 (intracellular program): Following OMV uptake and hypothesized endosomal release, BFT‐1 binds and stabilizes cytosolic NOD1, activating a GAK–NUMB–NOTCH1–HEY1 axis linked to stemness and chemoresistance. Arrows between tracks indicate an asymmetric feed‐forward relationship: Track 1 inflammatory output (IL‐6/JAK/STAT3) is proposed to reinforce Track 2 stemness programs, while Track 2 in turn amplifies the long‐term oncogenic consequences of junctional injury. Lower panel: working isoform‐bias hypothesis — BFT‐1, relative Track 2 bias; BFT‐2, relative Track 1 bias; BFT‐3, incompletely characterized. Solid lines, established [E]; dashed lines, evidence‐based synthesis [S]; dotted lines, falsifiable hypothesis [H]. Not drawn to scale. Created in biorender. Choi, K. (2026) https://BioRender.com/4tmnln2 (biorender publication license agreement number RY2AC9A3WC).

It is useful to situate this framework within the broader oncobiome field before developing it in detail. ETBF belongs to a small group of colorectal pathobionts for which causal, rather than merely associative, evidence exists, alongside *Fusobacterium nucleatum* (*F. nucleatum*) and pks^+^ *Escherichia coli* (*E. coli*). The three are instructive in their differences. pks^+^ *E. coli* acts principally through a direct genotoxin: repeated exposure of human intestinal organoids to pks^+^ bacteria generates a distinct mutational signature absent from organoids exposed to isogenic pks‐mutant controls, and the same signature is recoverable from human cancer genomes, predominantly colorectal [18]. *F. nucleatum*, by contrast, acts principally through adhesin‐mediated engagement of host receptors—FadA binds E‐cadherin and activates β‐catenin signaling [19], while Fap2 engages the inhibitory receptor TIGIT to suppress NK and T cell cytotoxicity [20] without a toxin‐attributable mutational signature. ETBF is distinguished by acting through a secreted protease whose immediate substrate is an adherens junction protein, E‐cadherin [4]. Its initiating lesion is thus the loss of junctional integrity rather than direct damage to DNA, and ETBF‐induced tumors accordingly carry no toxin‐attributable mutational signature (Section 6.1) [21]. This is not a claim that ETBF is non‐genotoxic: polyamine‐derived reactive oxygen species and sustained IL‐17–driven inflammation impose a well‐documented indirect mutational burden [22, 23]. The distinction is one of initiating lesion rather than of ultimate consequence.

The convergence is worth noting explicitly: Two unrelated colorectal pathobionts target the same host junctional protein by entirely different molecular strategies, one by non‐catalytic adhesin binding [19] and one by proteolytic cleavage of the ectodomain. This suggests that E‐cadherin is a recurrent point of microbial leverage on the colonic epithelium, and it is the reason why the topology of toxin action—the subject of this review—is mechanistically consequential in a way that it is not for a diffusible small‐molecule genotoxin.

Throughout what follows we distinguish three levels of confidence: claims that are established across laboratories and model systems, claims that represent our synthesis of existing evidence extended into a coherent framework, and claims that are explicitly hypothetical and stated so that they can be falsified. The surface program described in Section 2 falls almost entirely into the first category; the intracellular program of Sections 3 and 4, and the isoform track‐bias hypothesis of Section 5, fall substantially into the second and third.

## 2 The canonical surface program (Track 1)

The canonical model of ETBF‐mediated carcinogenesis posits that secreted BFT acts as an extracellular metalloprotease, initiating a self‐amplifying pro‐inflammatory cascade at the mucosal surface. Two points about this program should be stated at the outset, because they are easily conflated with the vesicular arguments developed in Section 3. First, Track 1 is defined by where BFT acts, not by how it arrived: claudin‐4‐dependent docking and E‐cadherin cleavage proceed identically whether the toxin reaches the receptor as free soluble protease or tethered to a vesicle surface, and the model developed here does not adjudicate between these routes. Second, essentially the entire experimental foundation of this program—including the receptor identification discussed below—was established using purified recombinant free toxin, and it is therefore free BFT that is demonstrably sufficient for every step of Track 1.

### 2.1 BFT biochemistry and E‐cadherin cleavage

BFT is synthesized as a pre‐pro‐enzyme and activated by the outer membrane protease fragipain [24]. The mature toxin's primary known epithelial substrate is E‐cadherin (CDH1) [4]. BFT cleaves the E‐cadherin extracellular domain 4 (EC4), generating a soluble 80 kDa ectodomain and disrupting homotypic trans‐adhesion [25]. Genetic ablation of *CDH1* in intestinal epithelial cells abolishes BFT‐induced IL‐8 secretion, positioning E‐cadherin shedding as the obligatory initiating event in the surface inflammatory program.

### 2.2 The β‐catenin and NF‐κB signaling axis

E‐cadherin cleavage disrupts the junctional tethering of β‐catenin, enabling its nuclear translocation [26]. Nuclear β‐catenin acts as a co‐activator of TCF/LEF‐dependent transcription and co‐regulates NF‐κB target genes [27]. This drives the transcription of a broad pro‐inflammatory program, including IL‐8 and CXCL1, establishing an acute inflammatory mucosal microenvironment [28]. Concurrently, BFT activates β‐catenin‐independent MAPK pathways, leading to AP‐1 activation, MMP‐7 induction, and syndecan‐2 shedding [28], which further remodels the pericellular matrix. Crucially, this NF‐κB‐dependent cytokine burst—particularly IL‐6 secretion—subsequently activates STAT3 in both a paracrine and autocrine fashion [29], establishing the critical inflammatory signaling node that will later converge with Track 2 epigenetic outputs [30, 31].

Importantly, biochemical evidence indicates that Track 1 is not a strictly linear cascade from E‐cadherin loss to NF‐κB activation [6]. Rather, specific functional assays demonstrate that BFT initiates at least two kinetically and mechanistically separable surface‐linked outputs: a junction‐centered arm, driven by E‐cadherin/β‐catenin release to amplify inflammatory transcription [32], and a parallel remodeling arm, where β‐catenin‐independent MAPK/AP‐1 signaling promotes MMP‐7 induction and syndecan‐2 shedding. This mechanistic bifurcation dictates that epithelial damage, cytokine release, and extracellular matrix remodeling need not occur with identical kinetics or to the same extent in every responsive cell type. Consequently, BFT exposure can produce widely different phenotypic balances depending on the specific junctional organization and signaling competence of the host cell—a heterogeneity that resonates directly with, and partially accounts for, the cell‐type‐selective BFT responsiveness (Paradox 1) introduced in Section 1.

### 2.3 STAT3 activation and EMT

STAT3 operates as a critical distal amplifier of the BFT inflammatory program. BFT activates the STAT3/ZEB2 signaling axis, enhancing cell migration, invasiveness, and hallmarks of epithelial‐to‐mesenchymal transition (EMT) [33]. STAT3 inhibition or ZEB2 knockdown reverses these phenotypic effects [34] and restores intestinal barrier integrity [35], identifying the STAT3/ZEB2 axis as a key effector of both EMT and barrier disruption.

### 2.4 Th17 immune amplification

The epithelial surface program is systemically amplified through the recruitment and activation of IL‐17A‐producing Th17 cells. Initial NF‐κB‐driven CXCL1 and IL‐8 secretion recruits immune effectors to the colonic mucosa [36]. Concurrently, regulatory T cells (Tregs) paradoxically promote tumorigenesis by consuming local IL‐2 [7]. This consumption suppresses Th1‐polarized anti‐tumor responses and permits the unchecked expansion of Th17 populations. Consequently, a self‐reinforcing mucosal immune amplification loop emerges, wherein IL‐17A, derived from redundant adaptive and innate sources, serves as the critical pro‐tumorigenic cytokine [37].

An essential implication of this immune circuitry is that tumor‐promoting inflammation during ETBF colonization is defined less by the presence of any single lymphocyte subset than by the persistence of an IL‐17‐dominant inflammatory microenvironment. This dynamic is powerfully illustrated by *in vivo* Treg depletion studies, which yield the apparently paradoxical result of exacerbated acute colitis coupled with significantly reduced tumorigenesis [7]. Furthermore, it clarifies why ETBF‐driven oncogenesis relies on chronic mucosal amplification rather than transient epithelial injury alone. Once established, the IL‐17/CXCL1/STAT3 signaling loop—sustained mechanistically by IL‐17A and IL‐6 driving JAK‐STAT3 phosphorylation in colonocytes—acts as a durable inflammatory scaffold that continuously reinforces epithelial survival, stromal recruitment, and downstream reprogramming pathways (discussed in Section 4) [37, 38], ultimately creating a permissive microenvironment where subsequent oncogenic outputs become progressively easier to sustain.

### 2.5 Epigenetic and metabolic reprogramming

Beyond acute inflammation, the surface program induces lasting oncogenic alterations [39]. Whole‐exome sequencing of ETBF‐driven tumors reveals lower rates of loss of heterozygosity at the *Apc* locus compared to spontaneous tumors [40], implicating alternative mutagenic mechanisms. Consistent with this, BFT exposure rapidly induces double‐strand DNA breaks within 3 h [41]. Furthermore, exposure over 24 to 48 h triggers widespread changes in chromatin accessibility that correlate with common differentially methylated regions found in CRC patients [42], directly bridging acute signaling with stable genomic landscapes. Metabolically, BFT hijacks host bile acid recycling to shift epithelial metabolism toward aerobic glycolysis. This generates a lactate‐ and oxygen‐rich niche that paradoxically supports the aerobic growth of ETBF, actively shaping the colonic microenvironment to favor persistent colonization.

These downstream effects fundamentally expand the significance of Track 1 beyond acute inflammatory signaling. The induction of DNA damage and altered chromatin accessibility suggests that BFT exposure leaves a durable molecular imprint even in cells that survive the initial insult [43], substantially lowering the threshold for subsequent clonal selection and malignant progression [44]. Likewise, the metabolic rewiring of the epithelium implies that ETBF does not merely respond to a pre‐existing dysbiotic niche, but actively constructs one. By promoting host glycolytic metabolism and altering luminal metabolite availability, BFT‐linked surface signaling generates a microenvironment that favors prolonged bacteria‐epithelial cohabitation. This creates a feed‐forward scenario in which early epithelial perturbation and microbial persistence become mutually reinforcing. Thus, even prior to the engagement of intracellular OMV‐mediated signaling, the canonical surface program already initiates long‐term niche engineering, linking initial barrier disruption to durable microenvironmental and epigenetic consequences that persistently drive tumor evolution [45].

### 2.6 Limits of the canonical model

Despite its substantial explanatory power, the surface proteolysis model fails to account for the cell type selectivity of BFT responsiveness, the topological paradox of cytosolic NOD1 engagement, or the functional non‐equivalence of BFT isoforms. Resolving these limitations requires an expanded framework focused on vesicular delivery.

## 3 OMV association as a candidate route to the intracellular compartment

BFT is released by ETBF in at least two physical forms: as a free, soluble metalloprotease, and as a toxin tethered to the surface of OMVs [46]. These forms are not mutually exclusive, and the quantitative partition between them in the colonic lumen *in vivo* has not been established. We emphasize that free BFT is fully sufficient for the surface program described in Section 2—claudin‐4‐dependent docking and E‐cadherin cleavage were demonstrated with purified recombinant toxin [16]—and nothing in the model developed here requires vesicular packaging for junctional injury to occur. OMV association is invoked for one specific and limited purpose: as a candidate mechanism by which BFT could reach a compartment that free toxin, now shown to act exclusively at the extracellular face of the plasma membrane [16], has not been shown to access.

### 3.1 ETBF OMVs and surface‐exposed BFT

ETBF OMVs carry a selectively enriched cargo that is spatially segregated [47, 48]. Nucleic acids, peptidoglycan, and specific lipoproteins reside within the vesicle lumen, where they are protected from extracellular degradation [17, 49]. By contrast, active BFT is localized to the outer vesicle surface. This outward‐facing topology has been unambiguously demonstrated by proteinase K degradation assays and liposome reconstitution studies [46]. The structural basis for this surface tethering involves complex interactions with the outer membrane, where hydrophobic grooves on the mature BFT surface accommodate fatty acid residues, and the catalytic zinc ion coordinates with phosphate oxygens [46]. Consequently, BFT is securely anchored in an orientation that permits direct interaction with epithelial substrates like E‐cadherin while remaining tethered to the vesicle [4, 46].

This topological arrangement is central to the plausibility of the two‐track model. If BFT were simply released as a freely diffusible protease, its biology would be largely restricted to extracellular substrate cleavage and short‐range surface signaling. In contrast, OMV association provides both concentration and context. It clusters the active toxin with other vesicular cargo and presents BFT in a membrane‐anchored orientation that is compatible with both initial epithelial surface engagement and subsequent intracellular trafficking following vesicle uptake. Accordingly, OMVs should not be viewed merely as a passive transport package for BFT. Rather, they constitute a highly organized delivery platform that fundamentally changes the spatial logic of toxin action. This mechanism provides a critical bridge between epithelial junctional cleavage at the cell surface and access to intracellular signaling compartments that would otherwise remain topologically inaccessible.

### 3.2 Endocytic uptake and cell type selectivity

The internalization of *Bacteroides* OMVs by host epithelial cells occurs primarily via dynamin‐dependent endocytosis and macropinocytosis [50]. However, the biological logic of ETBF cell type selectivity is now understood to operate through two molecularly distinct and independent layers—a surface layer anchored to claudin‐4 receptor expression and an intracellular layer governed by OMV endocytic competence—each gating a different functional program within the two‐track model.

#### 3.2.1 Layer 1: Claudin‐4‐mediated surface selectivity (Track 1 Gate)

The first selectivity layer—governing susceptibility to BFT‐mediated surface E‐cadherin cleavage—is now mechanistically defined. White et al. identified claudin‐4 (CLDN4) as the obligate BFT surface receptor through a genome‐wide CRISPR‐knockout screen and demonstrated that claudin‐4 expression level is the primary quantitative determinant of BFT responsiveness across epithelial populations [16]. Claudin‐4 single knockout in HT29 colonic epithelial cells confers approximately 20‐fold resistance to BFT‐mediated E‐cadherin cleavage, rising to approximately 500‐fold in claudin‐4/claudin‐3 double‐knockout lines; genetic rescue with GFP‐claudin‐4 fully restores sensitivity proportional to expression level [16]. Critically, the CRISPR screen hit list was dominated by plasma membrane‐localized proteins and secretory pathway components responsible for trafficking proteins to the cell surface, while known intracellular signaling proteins and host proteases were entirely absent [16]. This pattern establishes that the Layer 1 selectivity filter is exclusively a surface phenomenon: epithelia with low claudin‐4 expression cannot sustain productive BFT docking and are therefore resistant to Track 1 junctional injury, entirely independently of their E‐cadherin status or intracellular signaling capacity. The structural basis for this selectivity is provided by alphafold 3 modeling of the claudin‐4 : BFT : E‐cadherin ternary complex, in which claudin‐4 functions as a membrane‐localized scaffold that recruits BFT into an orientation poised to cleave the E‐cadherin linker region at residues Lys697‐Val701 [16]. Claudin‐3 can partially compensate for absent claudin‐4—as evidenced by the stepwise resistance increase from C4‐KO (20‐fold) to C4/C3‐DKO (500‐fold)—yet claudin‐3 constitutes a lower‐affinity receptor whose single amino acid difference at position T45 within extracellular segment 1 is sufficient to substantially impair BFT binding [16].

#### 3.2.2 Layer 2: OMV Endocytic competence as the Track 2 gate

The establishment of claudin‐4 as the sole determinant of Layer 1 selectivity carries a direct and important implication for the second selectivity layer: because the CRISPR screen explicitly excluded intracellular signaling proteins from the BFT surface‐activity pathway, and because NOD1 is a cytosolic receptor physically inaccessible to extracellular BFT, the Track 2 NOD1/GAK/NOTCH1 axis cannot be controlled by the claudin‐4 surface receptor logic. A second, independent selectivity filter must therefore operate at the intracellular level. We propose that this second layer is governed by the host epithelial cell's capacity for productive OMV internalization. Epithelia lacking the requisite membrane composition, lipid‐raft organization, or endocytic receptor profiles may fail to internalize ETBF OMVs efficiently, rendering them insensitive to OMV‐delivered BFT‐1 regardless of their claudin‐4 status and thereby refractory to Track 2 stemness reprogramming [51].

This Layer 2 hypothesis is further reinforced by the preferential enrichment of NOD1 expression in stem‐like epithelial populations. Even in cells with sufficient OMV uptake capacity, NOD1 abundance introduces a third, pathway‐internal selectivity gate: only NOD1‐high, stem‐like cells can mount a full GAK–NUMB–NOTCH1–HEY1 transcriptional response following OMV delivery. This creates an intrinsically tiered architecture in which OMV internalization is necessary but not sufficient for Track 2 engagement, with the final output calibrated by intracellular NOD1 abundance.

#### 3.2.3 The two‐by‐two selectivity matrix: A testable prediction

The independence of the two selectivity layers generates a falsifiable combinatorial prediction. Epithelial populations can in principle be classified into four functional states defined by the intersection of claudin‐4 expression (Layer 1) and OMV endocytic competence (Layer 2). Cells with high claudin‐4 expression and high OMV uptake capacity would undergo full two‐track engagement—experiencing both acute junctional disruption via Track 1 and sustained CSC expansion via Track 2—and represent the most oncogenically permissive state. Cells with high claudin‐4 but limited OMV uptake capacity would exhibit Track 1‐dominant responses: productive surface E‐cadherin cleavage with NF‐κB/STAT3/Th17 inflammatory amplification, but minimal stemness reprogramming. A third, mechanistically important state is predicted for cells with low claudin‐4 expression but intact OMV internalization: in these cells, surface BFT activity is largely absent (consistent with White et al.'s claudin‐4 KO data), yet OMV‐delivered BFT‐1 could, in principle, access cytosolic NOD1 to drive Track 2 programs in the relative absence of Track 1 junctional injury. Finally, cells with both low claudin‐4 expression and low OMV uptake capacity would remain largely refractory to ETBF pathobiology. This 2 × 2 matrix is directly testable: pharmacological inhibition of dynamin‐dependent endocytosis or macropinocytosis in claudin‐4‐competent cells should selectively abrogate Track 2 NOD1/CSC responses while leaving Track 1 E‐cadherin cleavage largely intact—a dissociation that would definitively confirm the independence of the two selectivity layers.

#### 3.2.4 An anatomical asymmetry further supports track independence

A fourth consideration supports the mechanistic independence of the two selectivity layers: the anatomical access routes by which free BFT and OMV‐associated BFT encounter the epithelial surface may not be identical. White et al. [16] propose that free BFT, secreted apically by ETBF colonizing the intestinal lumen, must traverse tight junctions to access basolateral claudin‐4 and E‐cadherin through an as‐yet‐uncharacterized mechanism, possibly exploiting cell extrusion sites or transient tight‐junction disruption. By contrast, OMVs, as small lipid‐bilayer vesicles, are capable of being endocytosed from the apical membrane surface via macropinocytosis—a process that is architecturally independent of tight‐junction integrity. This anatomical asymmetry implies that Track 1 and Track 2 are not only spatially distinct in their intracellular destinations but may also differ in the polarity of initial BFT acquisition. Consequently, OMV‐mediated Track 2 engagement could in principle precede or occur independently of the tight‐junction disruption that is prerequisite for free BFT to access its claudin‐4 receptor. This temporal and spatial flexibility broadens the oncogenically permissive window of Track 2 relative to Track 1, consistent with the observation that ETBF drives mesenchymal remodeling and establishes premetastatic niches in anatomical sites beyond the colon.

### 3.3 Intracellular cargo delivery and the topological paradox

Following endocytosis, a subpopulation of OMVs appears to exploit caveolin‐mediated pathways to bypass lysosomal degradation, trafficking directly to the host endoplasmic reticulum and Golgi apparatus. This internalization delivers luminal cargo (e.g., peptidoglycan and RNA) to intracellular compartments [52, 53, 54], activating pattern‐recognition receptors like NOD1 and TLR7 [55] that are physically inaccessible to non‐invasive bacteria [56].

Most importantly, the OMV delivery model resolves the topological paradox of intracellular BFT activity. When an ETBF OMV escapes lysosomal degradation, the outward‐facing topology of BFT on the OMV surface allows the toxin to directly interface with the host cytoplasm upon endosomal membrane fusion or disruption, providing unhindered access to cytoplasmic interaction partners like NOD1 [57].

### 3.4 Functional divergence: ETBF vs. NTBF OMVs

The presence of surface BFT fundamentally redefines the vesicle's biological program. While OMVs from non‐toxigenic *B. fragilis* (NTBF) typically deliver anti‐inflammatory signals via Polysaccharide A to promote tissue repair, ETBF OMVs simultaneously induce cell proliferation and apoptotic signaling. Thus, ETBF OMVs constitute a qualitatively distinct delivery system that effectively bridges the gap between extracellular junctional disruption and cytoplasmic pattern recognition.

## 4 Track 2: NOD1, stemness, and epigenetic memory

The activation of cytoplasmic receptors by internalized OMVs initiates Track 2, a signaling axis characterized by stemness induction and epigenetic reprogramming that operates independently of E‐cadherin cleavage.

### 4.1 The BFT‐1/NOD1/NOTCH1 cascade

Track 2 is defined by the direct engagement of cytosolic NOD1. Following OMV‐mediated intracellular delivery, BFT‐1 directly binds and stabilizes the NOD1 protein against proteasomal degradation—a physical interaction confirmed via co‐immunoprecipitation and LC–MS/MS in FLAG‐tagged NOD1‐overexpressing breast cancer cells. Crucially, NOD1 expression is not uniform across epithelial populations; it is preferentially enriched in stem‐like cells. This stabilized NOD1 forms a functional complex with cyclin G‐associated kinase (GAK) that directs NUMB phosphorylation [58, 59], targeting NUMB for lysosomal degradation. The depletion of NUMB removes the inhibitory brake on NOTCH1 processing [60], leading to the accumulation of the NOTCH1 intracellular domain (NICD1) [61] and the transcription of stemness genes like *HEY1* [62]. Consequently, this pathway selectively expands ALDH+ CSCs [63]. However, it is important to note that the NOD1‐binding specificity of BFT‐1 has been demonstrated primarily in breast cancer cells; direct head‐to‐head comparisons in colonic epithelial cells have not yet been established.

The significance of this pathway lies in the fact that it is supported by strict causal perturbation logic rather than correlative pathway mapping alone. For instance, GAK knockdown effectively reverses NOD1‐induced stemness phenotypes, and experimental NUMB rescue actively reverses NOTCH1 activation, confirming the obligate nature of these intermediate nodes. In this framework, NOD1 is not merely functioning as a canonical pattern‐recognition receptor for bacterial peptidoglycan [56], but as an intracellular signaling hub co‐opted to reprogram cell‐fate machinery. The downstream increase in ALDH‐positive CSCs, together with enhanced chemotherapy resistance—specifically taxane resistance in breast cancer models [64, 65]—places this axis squarely within a disease‐relevant oncogenic context. Just as importantly, the pathway helps explain why intracellular access matters: without OMV‐mediated delivery, the cytoplasmic NOD1 node would remain disconnected from extracellular BFT. Thus, Track 2 is not simply an extension of Track 1, but a qualitatively distinct program that depends on a different compartmental topology—intracellular rather than surface‐directed—a different receptor class, and a different set of biological outputs.

### 4.2 Epigenetic reprogramming: The JMJD2B/H3K9me3/NANOG axis

Operating in parallel to the cytosolic NOD1 cascade, ETBF also engages a surface‐directed signaling arm by selectively upregulating TLR4 (while leaving other membrane‐localized TLRs unaffected) and its downstream transcription factor NFAT5. This TLR4‐NFAT5 transcriptional axis drives the expression of the histone demethylase JMJD2B [66]. Once upregulated, JMJD2B actively removes repressive H3K9me3 marks from the *NANOG* promoter, inducing robust pluripotency factor expression [67]. The mechanistic necessity of this pathway is supported by strict perturbation logic: catalytically inactive JMJD2B mutants fail to demethylate the *NANOG* promoter [66], confirming the absolute requirement for enzymatic activity, while ectopic overexpression of NFAT5 or JMJD2B successfully rescues TLR4‐knockdown phenotypes. Crucially, the translational relevance of this axis is underscored by both *in vivo* and clinical data. JMJD2B knockdown significantly suppresses ETBF‐accelerated tumor growth in xenograft models, and CRC patient cohorts exhibit a strong positive correlation between ETBF abundance and the expression of NFAT5, JMJD2B, and NANOG. This arm warrants different evidential treatment from the NOD1 cascade described above, and the distinction is material to the objection that Track 2 is extrapolated from breast tissue. The TLR4–NFAT5–JMJD2B–NANOG axis was characterized in colorectal cancer cells, validated in CRC xenografts, and corroborated in CRC patient cohorts. It is therefore a colonic finding, independent of the breast cancer literature, and it establishes that ETBF exposure can drive stemness and epigenetic reprogramming in colonic epithelium through at least one demonstrated route. What remains unestablished in the colon is specifically the NOD1‐dependent route and its proposed dependence on intracellular BFT delivery.

This epigenetic arm is particularly important because it provides a credible molecular substrate for persistence. Whereas the NOD1/NOTCH1 cascade can account for the rapid, acute expansion of stem‐like populations [68], the JMJD2B‐centered pathway explains how transient ETBF exposure converts into a more durable transcriptional state—a phenomenon termed “BFT memory.” By eroding repressive H3K9me3 marks at pluripotency‐associated loci, ETBF‐linked signaling creates a chromatin landscape in which stemness programs become easier to re‐induce and harder to extinguish [66]. In this sense, Track 2 contains both an acute signaling component and a memory‐forming epigenetic component, functionally linking initial microbial exposure to persistent oncogenic states that can ultimately outlast the pathogen itself.

### 4.3 “BFT memory” and chemoresistance

This durable epigenetic reprogramming underpins the “BFT memory” phenomenon, wherein brief exposure to BFT elicits persistent CSC expansion, EMT, and enhanced metastatic potential that vastly outlasts the bacterial trigger [69]. Clinically, this track confers profound chemoresistance. The BFT‐1/NOD1 axis mediates taxane resistance in breast cancer models, while similar NOTCH1‐driven pathways mediate 5‐FU and oxaliplatin resistance in CRC [70, 71], positioning Track 2 as a major driver of treatment‐refractory disease. Single‐cell RNA sequencing further confirms that ETBF induces CSC‐like transit‐amplifying cell populations while simultaneously suppressing anti‐tumor cytotoxic T‐lymphocyte functions [72].

### 4.4 Convergence and persistence

Tracks 1 and 2 operate as a highly coordinated, feed‐forward loop. The acute mucosal inflammation and IL‐17 output from Track 1 robustly activate STAT3, which in turn acts as a transcriptional pivot to reinforce the JMJD2B‐mediated epigenetic reprogramming of Track 2. Ultimately, the transient barrier disruption of Track 1 is “locked in” by Track 2's epigenetic memory, generating a stable, chemoresistant cancer stem cell niche. This synergistic convergence explains how acute bacterial exposure translates into durable, self‐sustaining oncogenic progression independently of persistent colonization.

## 5 Structural diversity of BFT isoforms and the track‐bias hypothesis

Three decades of molecular epidemiology have yielded a persistent paradox: strains encoding *bft‐1* predominate in adult CRC, whereas *bft‐2* strains are disproportionately recovered from pediatric diarrheal disease. We propose that this epidemiological asymmetry reflects intrinsic structural differences in the catalytic domains of BFT‐1 and BFT‐2 that create a systematic functional bias.

### 5.1 The three‐isoform system and catalytic divergence

The three BFT isoforms share greater than 90% overall amino acid identity, possessing a conserved pro‐domain and a shared metzincin catalytic core (HEXXHXXGXXH). Globally, *bft‐1* is the most prevalent allele (~ 65% of human isolates) and is heavily enriched in CRC. Conversely, the *bft‐2* allele is significantly enriched in acute pediatric diarrhea (*P* = 0.0064) [15].

Crucially, while the metzincin core is invariant, the surface loops surrounding the active‐site cleft exhibit discrete substitutions. Residue 357, located within the substrate‐guiding upper‐rim strand of the zinc‐binding motif, represents one of the most pronounced points of divergence between BFT‐1 and BFT‐2 [73]. Variation at this specific position is predicted to directly influence the geometry of the substrate‐binding cleft, producing isoforms with quantitatively distinct substrate affinities rather than a simple binary switch (Fig. 2A).

**Fig. 2:**
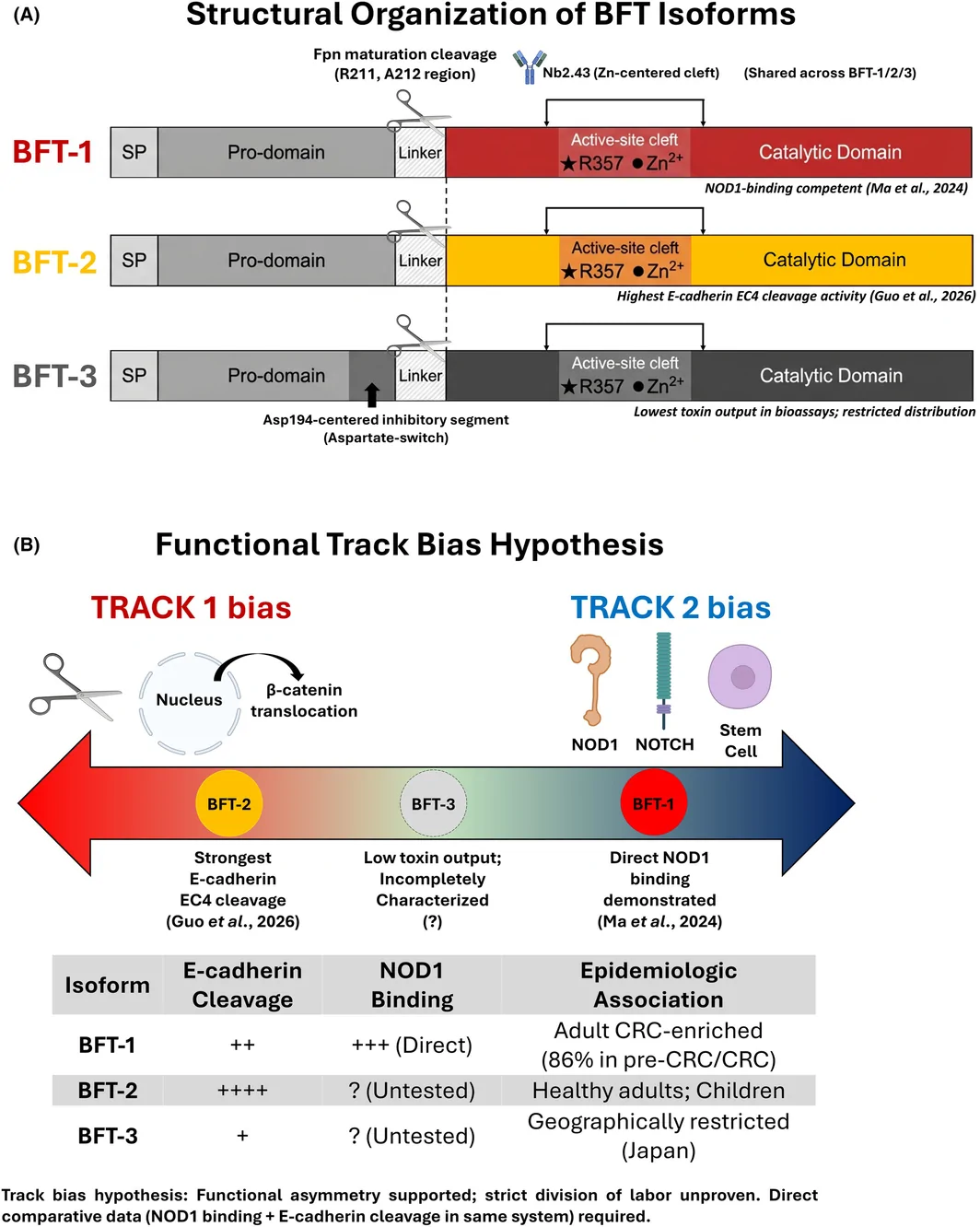
Structural organization and functional track‐bias hypothesis of BFT isoforms. (A) Schematic comparison of BFT‐1, BFT‐2, and BFT‐3 preprotein domain architecture, comprising signal peptide (SP), pro‐domain, linker/maturation boundary, and catalytic domain. Scissors denote Fpn (fragipain)‐mediated pro‐domain removal required for zymogen activation; isoform‐specific differences near the cleavage junction may influence maturation kinetics but have not yet been directly compared across all three isoforms. A star within the catalytic domain marks residue 357, identified as a key determinant of subtype‐specific activity differences between BFT‐1 and BFT‐2. The Zn^2+^ label indicates the catalytic zinc‐containing active‐site cleft; the bracketed region denotes the Nb2.43 binding site, through which this nanobody neutralizes all three isoforms by direct catalytic‐site engagement and zinc chelation. In BFT‐3 only, a compact segment adjacent to the pro‐domain/catalytic domain boundary is annotated as the aspartate‐switch inhibitory element, which maintains zymogen latency prior to activation; the residue designation is based on the primary structural literature for fragilysin‐3 and is not annotated for BFT‐1 or BFT‐2 at equivalent resolution. The dashed vertical line marks the approximate maturation cleavage region and does not indicate the position of residue 357. Domain boundaries are approximate; not drawn to scale. (B) Working model of proposed functional asymmetry across BFT isoforms relative to the two‐track oncogenic framework; this panel represents a bias hypothesis, not a strict or proven division of labor. Left axis: relative bias toward Track 1 (surface E‐cadherin program; junctional injury, β‐catenin signaling, inflammatory output). Right axis: relative bias toward Track 2 (intracellular program; NOD1 engagement, NOTCH1 signaling, stemness, chemoresistance). BFT‐2 is positioned toward Track 1 on the basis of the highest reported E‐cadherin EC4 cleavage activity among the three isoforms. BFT‐1 is positioned toward Track 2 on the basis of directly demonstrated NOD1 binding and stabilization in breast cancer stem‐like cells, with downstream activation of a GAK–NUMB–NOTCH1–HEY1 stemness program. BFT‐3 is placed centrally as incompletely characterized, reflecting lower biologically active toxin output in historical isolate studies and insufficient mechanistic definition. The accompanying table summarizes relative E‐cadherin cleavage activity, NOD1 binding status, and representative epidemiologic associations—*bft‐1* enriched in adult and CRC‐associated isolates; *bft‐2* in healthy and pediatric isolates; *bft‐3* geographically restricted with low toxin output—for each isoform. These assignments should be updated as direct head‐to‐head isoform comparison data become available. Created in BioRender. Choi, K. (2026) https://BioRender.com/e0kdvz8 (BioRender publication license agreement number XP2AC99NXP).

### 5.2 Structural determinants of track bias

The recent identification of claudin‐4 as the obligate BFT surface receptor [16] establishes that the initial membrane‐docking step is, at minimum, partially shared across isoforms that retain the relevant binding geometry. This implies that receptor‐level differences cannot account for the isoform‐dependent divergence in oncogenic programs; rather, track bias must arise from post‐receptor‐binding events, focusing mechanistic attention squarely on the catalytic domain itself.

The structural translation of these sequence differences—most critically the divergence at residue 357 within the substrate‐guiding upper‐rim strand—manifests in distinct catalytic kinetics and membrane interactions. Comparative biochemical characterization reveals that BFT‐2 demonstrates significantly higher biological activity in epithelial models (*P* ≤ 0.01), inducing E‐cadherin ectodomain shedding more efficiently than BFT‐1 [73]. Mechanistically, this superior cleavage velocity is structurally supported by an amphipathic region at the carboxyl terminus of the mature BFT‐2 protein, which facilitates sustained interaction with the host cell plasma membrane—a feature that is non‐equivalent in BFT‐1 [74]. These biochemical and structural properties position BFT‐2 as the isoform optimized for maximal, rapid E‐cadherin proteolysis at the epithelial surface, aligning perfectly with the acute barrier disruption and robust inflammatory secretion characteristic of the Track 1 program.

In contrast, the Track 2 bias of BFT‐1 is driven by its unique capacity for intracellular NOD1 engagement [75]. The isoform‐specificity of this interaction suggests that discrete surface residues in the catalytic domain of BFT‐1—likely governed by the local geometry around residue 357—create a topographic complementarity with the leucine‐rich repeat domain of NOD1 that is absent or attenuated in BFT‐2. Crucially, in the absence of an E‐cadherin‐like substrate in the intracellular environment, BFT‐1's specific surface geometry may permit a stable, non‐proteolytic docking interaction with NOD1. The higher E‐cadherin cleavage velocity of BFT‐2, meanwhile, may direct its activity predominantly toward the extracellular substrate, limiting the fraction that productively traffics to engage intracellular targets.

Although the track‐bias model is most compelling when framed as a contrast between BFT‐1 and BFT‐2, the behavior of BFT‐3 is also conceptually informative. Characterized as a conditionally disordered protein heavily dependent on zinc coordination, BFT‐3 exhibits consistently low active toxin output. Its zymogen is held latent by an unusually large pro‐domain that inhibits the catalytic domain through an aspartate‐switch mechanism and also contributes to folding stability [76], and its maturation can be blocked allosterically by small molecules that shift a zinc‐dependent disorder‐to‐order equilibrium, stabilizing a partially unstructured, zinc‐free conformation incompetent for autoactivation [77]. Its stronger dependence on structural stabilization suggests that the fragilysin family is not simply divided into “oncogenic” and “non‐oncogenic” variants, but instead spans a spectrum of catalytic stability and biological deployment. This perspective strengthens the central hypothesis by showing that isoform diversity is biologically meaningful even when it does not produce equally strong disease associations. At the same time, it underscores an important caveat: the NOD1‐binding specificity of BFT‐1 has been demonstrated primarily in breast cancer cells; direct head‐to‐head comparisons of BFT‐1 versus BFT‐2 for NOD1 engagement in colonic epithelial cells have not yet been established. Accordingly, the track‐bias hypothesis rests on an isoform‐level inference drawn substantially from a non‐colonic system, and head‐to‐head testing of BFT‐1 against BFT‐2 for NOD1 engagement and E‐cadherin cleavage in colonic epithelial cells is a prerequisite for the hypothesis to be regarded as more than a hypothesis. Accordingly, the present model should be interpreted as an evidence‐based hierarchy rather than a finalized classification scheme, with the strongest current support favoring differential Track 1 versus Track 2 bias between BFT‐2 and BFT‐1, respectively (Fig. 2B).

### 5.3 Synthesis: The track‐bias hypothesis

Synthesizing these findings, we propose a functional track‐bias model:

- *BFT‐2 (Track 1 Predominance)*: Optimized for extracellular E‐cadherin cleavage, driving acute mucosal barrier disruption and immune recruitment.
- *BFT‐1 (Track 2 Predominance)*: Structurally equipped for direct intracellular NOD1 engagement, driving CSC expansion, epigenetic memory, and chemoresistance—aligning with its prevalence in adult CRC.

This framework resolves the longstanding paradox of isoform‐dependent disease associations by linking subtle structural differences on the BFT catalytic surface to profound functional divergences in oncogenic trajectories. However, rigorous crystallographic characterization of BFT‐1/NOD1 binding interfaces and head‐to‐head comparisons in colonic epithelial cells remain necessary to fully validate this hypothesis.

### 5.4 Reinterpreting epidemiological heterogeneity through the track‐bias lens

If the track‐bias hypothesis is correct, it carries an immediate implication for how three decades of ETBF epidemiology should be read. Reported associations between ETBF colonization and colorectal cancer have varied considerably across studies and geographical regions, and this variability has generally been treated as inconsistency to be resolved by larger samples. The model proposed here suggests an alternative reading: that a substantial part of the heterogeneity is structural, arising because studies have measured different exposures while reporting them under a single label.

Three sources of structural heterogeneity follow directly from the model. The first is isoform composition. Most published studies report only total *bft* positivity, yet the relative prevalence of the three alleles differs markedly between isolate collections. bft‐1 predominates in most surveyed populations [15], while *bft‐3* was first identified in Japanese and Korean isolates [78, 79] and remains largely confined to East Asian collections. Under the track‐bias hypothesis, two cohorts with identical total *bft* prevalence may carry substantially different Track 1 to Track 2 output and would therefore be expected to associate with different clinical endpoints: a *bft‐2*‐dominant population more strongly with acute inflammatory and mucosal‐injury phenotypes, a *bft‐1*‐dominant population more strongly with advanced, stemness‐high, chemoresistant disease. Pooling these into a single ETBF‐positive stratum would predictably attenuate and destabilize effect estimates.

The second is the sampling compartment. Stool, mucosal biopsy, and tumor tissue do not interrogate the same exposure. Because the surface program requires BFT to reach basolateral claudin‐4 and the proposed intracellular program requires vesicle endocytosis at the epithelial surface, mucosa‐adherent and biofilm‐associated ETBF is mechanistically far more informative than luminal carriage. This is not merely a sampling technicality: ETBF is one of two organisms found to dominate the mucus‐embedded biofilms of familial adenomatous polyposis patients [80], placing it squarely in the compartment where both tracks could operate. Stool‐based detection, which dominates the larger studies, therefore has systematically weaker discriminative power for precisely the mechanistic reasons the model articulates. The available isoform data illustrate the resulting limitation: in one stool‐based series, *bft‐1* predominated almost equally among colorectal cancer patients and controls [8], a result that is uninformative about track bias because neither compartment nor endpoint was resolved.

The third is detection endpoint. Gene detection, toxin protein detection, and serology capture different time windows. Serological studies capture cumulative historical exposure, which is the exposure metric most compatible with a model incorporating durable epigenetic memory; cross‐sectional PCR captures only concurrent colonization. A framework in which transient exposure produces persistent oncogenic states predicts exactly the pattern observed in the literature—relatively consistent pre‐diagnostic serological associations [11] alongside more variable cross‐sectional detection results [2]—and this pattern is difficult to interpret under a model requiring continuous colonization for continuous effect.

These considerations generate a concrete and inexpensive recommendation. Retrospective isoform‐resolved genotyping of existing biobanked ETBF isolate collections, stratified by sampling compartment and clinical endpoint, would test the track‐bias hypothesis directly without requiring new cohort recruitment. We further suggest that future studies report isoform genotype, sampling compartment, and detection modality as minimum metadata. We stress that isoform composition is unlikely to explain all reported heterogeneity: host genetics, diet, co‐colonizing taxa including *F. nucleatum* and pks^+^ *E. coli*—the latter co‐dominant with ETBF in polyposis biofilms [80]—and differing screening intensity plainly contribute. The claim is narrower: that isoform composition is a currently unmeasured and mechanistically motivated source of variance, and that measuring it is cheap.

## 6 Integrated model, clinical implications, and key unresolved questions

The preceding sections have characterized Track 1 (surface‐directed inflammation) and Track 2 (intracellular stemness) as distinct outputs of BFT signaling. However, in the context of chronic colonization, these tracks operate as an integrated, feed‐forward system that drives aggressive tumor phenotypes and demands a reimagined therapeutic approach (Fig. 3).

**Fig. 3:**
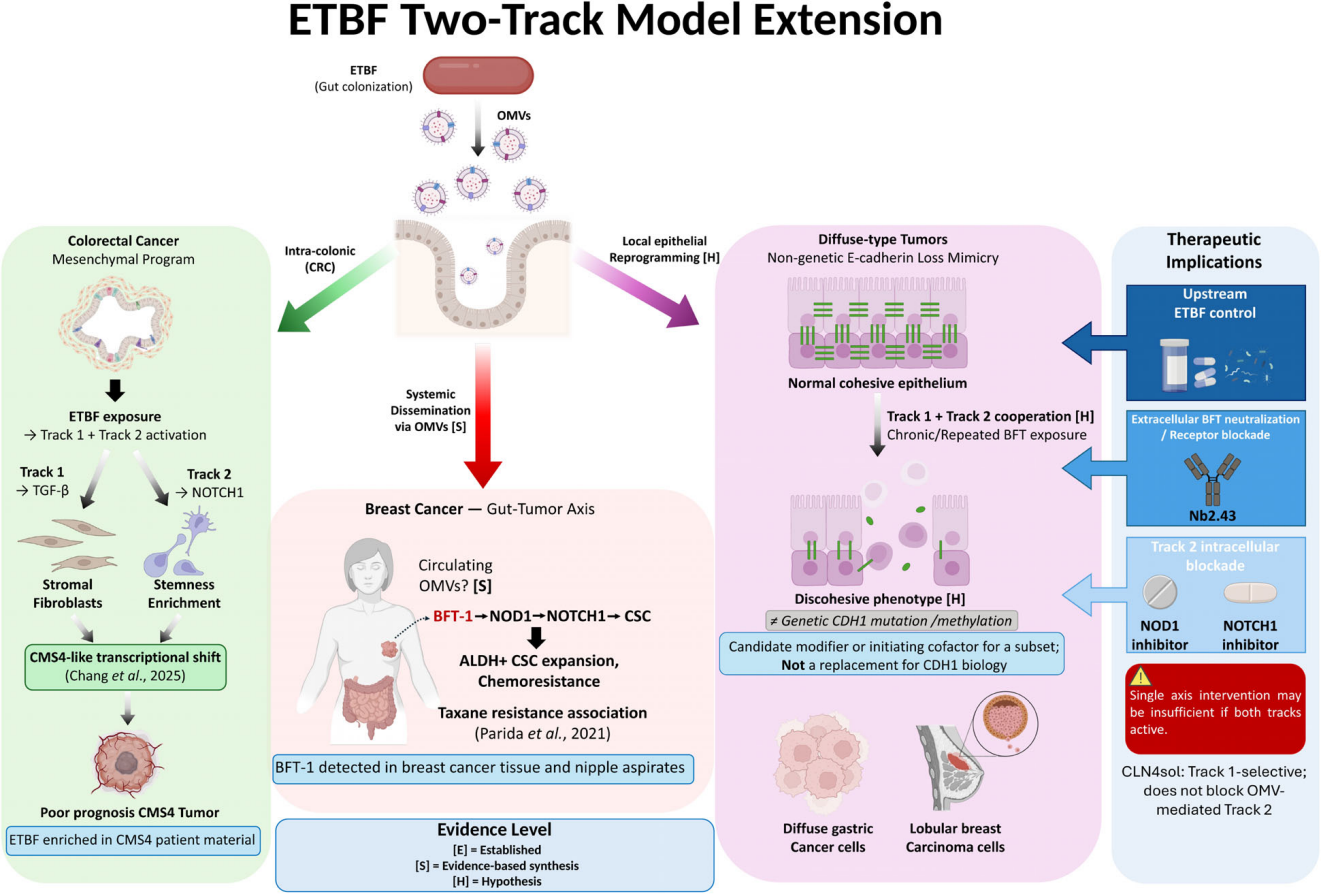
Extension of the ETBF two‐track model to mesenchymal CRC, extra‐colonic gut–tumor signaling, diffuse‐type epithelial tumors, and layered therapeutic implications. Conceptual extension of the two‐track framework; established observations [E], evidence‐based synthesis [S], and explicit hypothesis [H] are distinguished throughout; several depicted mechanistic links remain inferential. CRC/CMS4 panel: joint Track 1 (inflammatory and stromal) and Track 2 (stemness) activation is proposed to facilitate a CMS4‐like mesenchymal transcriptional shift, supported by patient‐derived enrichment data and CMS2‐organoid modeling; ETBF is depicted as a microbial facilitator, not the sole CMS4 driver [S/H]. Breast cancer/gut–tumor axis panel: OMV‐associated ETBF factors are proposed as candidate mediators of distal epithelial influence; BFT‐1–NOD1–NOTCH1 signaling is linked to cancer stem‐like cell expansion and an observational association with taxane resistance (causality not established; depicted as [S/H]); systemic OMV delivery to breast tissue remains a mechanistic hypothesis [H]. Diffuse‐type tumor panel: combined Track 1 and Track 2 ETBF exposure is proposed to generate a non‐genetic E‐cadherin‐loss‐like discohesive phenotype; ETBF is framed as a candidate modifier or initiating cofactor, not a substitute for canonical CDH1‐inactivating biology [H]. Therapeutic panel: layered strategy comprising upstream ETBF control, extracellular BFT neutralization (including active‐site blockade via Nb2.43 and Track 1‐selective claudin‐4 receptor blockade via CLN4sol [E]), and Track 2 intracellular blockade; CLN4sol inhibits claudin‐4‐dependent surface BFT activity *in vivo* but does not impede OMV‐mediated intracellular delivery, underscoring that receptor blockade alone cannot substitute for Track 2‐directed intervention. Single‐axis intervention may be insufficient when both tracks are concurrently active. Not drawn to scale. Created in BioRender. Choi, K. (2026) https://BioRender.com/xdvhlri (BioRender publication license agreement number LD2AC9AEGH).

### 6.1 Feed‐forward convergence and morphological extensions

The central synergy of the two‐track model is that Track 1 inflammatory outputs actively prime the cellular niche for Track 2 epigenetic reprogramming through three distinct mechanisms. First, Treg‐mediated immunosuppression suppresses IFN‐γ‐driven anti‐tumor surveillance [81], effectively removing the immunological pressure that would otherwise eliminate early CSC clones generated by Track 2. Second, the Treg‐amplified IL‐17 milieu phosphorylates STAT3 via the IL‐17R–Act1–TRAF6–NF‐κB axis [82]. This maintains the transcriptional environment required for JMJD2B upregulation independently of ongoing BFT cleavage events. Third, BFT‐induced metabolic reprogramming toward glycolysis creates a lactate‐ and oxygen‐rich microenvironment that favors ETBF oxidative metabolism, establishing a positive feedback loop for chronic toxin secretion.

Within this primed environment, STAT3 serves as the master molecular pivot. It not only sustains the pro‐inflammatory cytokine output but also upregulates the ZEB2 transcription factor to drive EMT and activate a TGF‐β1 autocrine signaling loop [83, 84]. Concurrently, STAT3 reinforces the TLR4–NFAT5–JMJD2B axis necessary for Track 2 NANOG‐dependent stemness [85]. The ultimate endpoint of this convergence is a striking molecular resemblance to the Consensus Molecular Subtype 4 (CMS4) of CRC. CMS4 represents approximately 23% [86] of CRC cases and is characterized by profound stromal invasion, marked TGF‐β activation, and the worst overall and relapse‐free survival among all subtypes [87, 88, 89, 90].

This integrated pathogenic program extends actively into the metastatic niche and extra‐colonic tissues. Droplet digital PCR analyses have detected ETBF DNA in the vast majority of CRC liver metastasis specimens [91], where higher ETBF DNA levels correlate with a microenvironment enriched in CD68^+^ macrophages and depleted in FOXP3^+^ Tregs—a pattern consistent with M2‐polarized, immune‐evasive remodeling [92]. Furthermore, recent evidence defines an ETBF‐orchestrated Gut–Breast Axis [69], wherein gut colonization generates systemic IL‐17 elevation that primes premetastatic niches in the liver and lungs [12], while circulating BFT‐1 directly engages NOD1 on breast cancer stem cells to confer taxane resistance [75]. Thus, ETBF acts as a systemic oncogenic driver whose organ‐tropism is dictated by the receptor expression profile of the target epithelium and the specific *bft* isoform present.

Viewed in this way, the value of the two‐track model is not merely that it explains multiple observations at once, but that it organizes them temporally and spatially. Track 1 is likely to dominate early, when surface‐accessible BFT rapidly disrupts junctions and initiates cytokine release. Track 2 emerges progressively as OMV uptake, intracellular delivery, and chromatin remodeling accumulate over time. This staggered architecture helps explain how ETBF can be enriched in precancerous lesions yet remain mechanistically relevant in advanced, therapy‐resistant tumors. It also clarifies why the clinical phenotype is not uniform across all ETBF‐positive cancers: some lesions may be dominated by inflammatory‐mesenchymal outputs, whereas others may be more strongly shaped by stemness, epigenetic persistence, and immune evasion. Framed this way, the two‐track model offers a way to think about ETBF not as a single oncogenic event, but as a dynamic system whose dominant outputs shift with host context, tumor stage, and isoform composition. Tracks 1 and 2 therefore operate as a feed‐forward loop rather than as isolated pathways (Fig. 1).

#### 6.1.1 Anatomical and molecular subtype granularity: where should each track dominate?

The model as developed so far applies granularity to the microbial side—isoforms, physical forms, delivery routes—while treating the host epithelium as comparatively uniform. Colorectal cancer is not uniform, and the model gains both plausibility and testability when the same resolution is applied to host context.

Proximal versus distal colon: an instructive tension. The two requirements for Track 1 are anatomically discordant. On the receptor side, claudin‐4—together with claudins‐3, ‐7 and ‐8—is predominantly expressed in the distal gastrointestinal tract, with lower expression proximally [93, 94]; Layer 1 (Track 1 gate) permissiveness is therefore distally weighted. On the access side, the situation is reversed: dense mucus‐embedded bacterial biofilms in direct epithelial apposition are nearly universal on right‐sided colorectal tumors and are found on only a small minority of distal ones [95], and ETBF is among the organisms that dominate such biofilms [80]. Because free BFT secreted apically must traverse the tight junction to reach basolateral claudin‐4 [16], Track 1 requires the coincidence of receptor availability and barrier breach, and neither region supplies both optimally.

This tension is productive rather than problematic. It predicts that Track 1 engagement should be patchy and focal rather than regionally uniform, concentrated wherever biofilm‐mediated epithelial contact and adequate claudin‐4 expression happen to overlap, and it offers an explanation for why ETBF‐associated tumors are not confined to a single colonic segment despite the strongly proximal distribution of biofilms. Track 2 is subject to a different geometry altogether: apical vesicle endocytosis is architecturally independent of tight‐junction integrity and of claudin‐4 expression, so the proposed intracellular program could in principle remain operative in distal segments where biofilm‐mediated access is scarce, and in proximal segments where claudin‐4 is comparatively low. The two tracks are thus predicted to dissociate anatomically as well as mechanistically—a prediction directly testable by regionally resolved sampling.

APCmin versus BRAFV600E models. The two principal murine systems used to establish ETBF tumorigenesis are not interchangeable, and our mechanistic claims should be attributed to the system that generated them. The classic ETBF tumorigenesis data derive from APCmin/+ mice, in which tumors are distal, Wnt‐driven, and microsatellite‐stable; Track 1 outputs map most naturally onto this system. Notably, whole‐exome and whole‐genome sequencing of ETBF‐induced tumors in this model shows that ETBF, unlike pks^+^ *E. coli*, leaves no unique mutational signature: tumors arise through the same Apc LOH mechanism that accounts for sham tumors, with the route shifting to acquired protein‐truncating Apc mutations only when mismatch repair is disrupted [21]. This is what a model of toxin‐ and inflammation‐driven promotion, rather than direct mutagenesis, would predict. BRAFV600E‐driven models recapitulate the serrated, proximal, frequently CIMP‐high pathway, in which MLH1 promoter methylation drives progression toward deficient mismatch repair (dMMR)/microsatellite‐instability–high (MSI‐H) disease [96], and notably ETBF‐driven tumorigenesis in this system is sensitive to immune checkpoint therapy [3]. We suggest that the serrated axis is the more natural host context for the proposed intracellular program, because the serrated pathway is itself defined by epigenetic silencing and stem‐like crypt phenotypes—the precise substrate on which a JMJD2B/H3K9me3/NANOG memory arm and a NOTCH1‐linked stemness arm would be expected to act. This yields a falsifiable prediction: isoform‐matched ETBF challenge in APCmin/+ versus BRAFV600E models should produce divergent track signatures, with bft‐2 disproportionately potentiating the former and *bft‐1* the latter.

dMMR versus proficient mismatch repair (pMMR). The mismatch‐repair dependence just described is not incidental—the same sequencing study shows that host repair status determines which genetic route ETBF‐associated tumors take [21]—and we propose that the two tracks segregate across that same axis. The surface program is an inflammatory‐mesenchymal program whose convergent endpoint we identify as CMS4, a subtype that is predominantly mismatch‐repair‐proficient and TGF‐β‐high; we therefore predict that *bft‐2*‐biased, Track 1‐dominant exposure will be enriched in pMMR CMS4 disease. Mismatch‐repair‐deficient tumors present a different calculus. They arise disproportionately in the proximal colon along the CIMP/BRAF/serrated axis, are hypermutated, and are immunologically hot. In a hypermutant background, toxin‐driven DNA damage is less likely to be rate‐limiting, whereas the epigenetic‐silencing background makes the memory arm potentially more consequential; and the Treg/IL‐17 circuitry of Track 1 becomes relevant chiefly as a determinant of immune evasion and checkpoint‐inhibitor response rather than as a mutagenic driver. This last point carries a direct translational read‐out. First‐line PD‐1 blockade is now standard of care in dMMR/MSI‐H metastatic colorectal cancer, yet a substantial minority of patients progress immediately, indicating primary resistance that mismatch repair status alone does not predict [97]. ETBF status and isoform therefore warrant examination as covariates in these cohorts.

### 6.2 Track‐stratified therapeutic framework

The two‐track framework dictates that single‐axis interventions will be insufficient to arrest ETBF‐driven carcinogenesis, necessitating a precision medicine roadmap organized into complementary tiers.

*Diagnostic stratification*: Effective intervention requires advancing beyond total *bft* detection to isoform‐resolved genotyping. Distinguishing *bft‐1*‐dominant carriers (at risk for NOD1‐mediated chemoresistance) [98] from *bft‐2*‐dominant carriers (at risk for Track 1‐driven mesenchymal CRC) is a critical prerequisite for biomarker‐stratified clinical trials [99].

*Precision eradication vs. broad antibiotics*: ETBF exhibits significant resistance to metronidazole, clindamycin, and multiple β‐lactams [100]. More importantly, the tumor‐suppressive versus tumor‐promoting duality of *Bacteroides fragilis* strictly depends on its virulence gene content [101]. NTBF, particularly when enhanced by vitamin D signaling, acts as an immunostimulatory commensal that significantly augments immune checkpoint inhibitor (ICI) responses [102]. Conversely, ETBF actively suppresses anti‐tumor immunity via CD68^+^ macrophage polarization, actively undermining ICI efficacy [91]. Consequently, broad‐spectrum antibiotics risk depleting the beneficial NTBF populations [103]. This paradigm emphasizes the absolute necessity of ETBF‐selective eradication—such as the targeted bacteriophage VA7, which preserves ICI‐supportive commensal ecosystems—prior to therapeutic deployment.

*Track 1‐targeted receptor blockade*: The identification of claudin‐4 as the obligate BFT surface receptor [16] introduces a mechanistically novel therapeutic entry point. CLN4sol—a soluble claudin‐4 analogue displaying the native extracellular segments—competitively blocks BFT binding to colonic claudin‐4, inhibiting E‐cadherin cleavage *in vitro* and significantly reducing epithelial cell shedding and edema in a mouse cecal injection model [16]. Because CLN4sol acts specifically at the claudin‐4 : BFT interface rather than targeting the conserved metalloprotease active site, it avoids the musculoskeletal off‐target toxicity that has hindered broad metalloprotease inhibitor development. Importantly, claudin‐4 blockade is inherently Track 1‐selective: it would not impede OMV‐mediated intracellular BFT‐1 delivery and therefore cannot substitute for Track 2‐directed interventions in bft‐1‐dominant disease contexts.

*Track‐specific co‐intervention*: Because the epigenetic “BFT memory” persists independently of ongoing bacterial colonization, eradication alone cannot fully reverse established oncogenic states. Pre‐clinical models demonstrate that NOTCH1 blockade alone is insufficient for full chemosensitivity without concurrent bacterial clearance, while NOD1 inhibition alone provides only partial chemosensitization. Therefore, phage therapy must be coupled with track‐specific pharmacological interventions [104]. These include STAT3 inhibitors (e.g., TTI‐101, ponatinib) to decouple the CMS4 inflammatory‐mesenchymal feed‐forward loop [105], and Track 2‐targeted agents (such as direct NOD1 inhibitors) to dismantle the chemoresistant stem cell compartment. The lack of clinically validated JMJD2B/KDM4B inhibitors with colonic epithelial activity remains a critical gap in this therapeutic pipeline.

*Chemoprevention*: For high‐risk ETBF‐colonized individuals (e.g., IBD patients or familial cohorts), dietary and nutraceutical interventions targeting the NF‐κB/IL‐17 inflammatory axis (such as caffeic acid phenethyl ester) represent a complementary, low‐toxicity preventative strategy [106].

This therapeutic logic has an important practical implication: eradication and pathway blockade should be viewed as complementary rather than competing strategies. If ETBF contributes to tumor progression primarily through ongoing Track 1 inflammatory stimulation [107], then selective bacterial clearance may be sufficient in early‐stage or high‐risk premalignant settings. However, if ETBF has already established a stable Track 2 program marked by stemness, chemoresistance, and epigenetic memory, then bacterial eradication alone is unlikely to reset the system [66, 69]. In that setting, host‐directed co‐intervention becomes essential. This is precisely why isoform‐resolved diagnostics may matter clinically: a lesion dominated by Track 1‐like outputs may be more responsive to anti‐inflammatory or anti‐STAT3 strategies [108], whereas a lesion with stronger Track 2 signatures may require direct disruption of NOD1/NOTCH1‐linked stemness or JMJD2B‐associated epigenetic maintenance. Thus, the translational value of the model lies not only in identifying ETBF as a target but in encouraging a staged, biologically stratified treatment framework.

### 6.3 Priority experimental agenda

While the two‐track model provides a coherent organizational framework, several mechanistic assertions require direct experimental validation, and the major testable predictions are summarized in Table 1:

- *Structural basis of track bias*: High‐resolution cryo‐EM co‐crystallization of BFT‐1/NOD1 and BFT‐2/E‐cadherin complexes is essential to unambiguously confirm whether the residue 357 polymorphism dictates differential receptor engagement. This structural resolution is also a prerequisite for designing cross‐isoform allosteric BFT inhibitors.
- *Epigenetic validation*: The specific requirement of the JMJD2B/KDM4B axis as the mediator between STAT3 activation and NANOG‐dependent stemness memory must be validated via epigenetic mapping in primary ETBF‐infected tumor models, with and without STAT3 or KDM4B inhibition.
- *Spatial track dominance*: Spatial transcriptomics of ETBF‐colonized tumors is needed to rigorously test whether track bias produces spatially segregated oncogenic niches (e.g., Track 1‐marked pSTAT3^+^ zones versus Track 2‐marked NOD1^+^ zones) within individual tumors.
- *Isoform‐resolved epidemiology*: Retrospective genotyping of banked ETBF isolate collections for *bft* allele, stratified by sampling compartment (stool, mucosal biopsy, tumor) and by clinical endpoint, is required to test whether the track‐bias hypothesis accounts for a measurable fraction of the heterogeneity in reported ETBF–CRC associations. This is the lowest‐cost test of the model available, as it requires no new cohort recruitment.

**Table 1:** Priority experiments for testing the OMV‐associated two‐track model of ETBF carcinogenesis. Priority experiments proposed to test the OMV‐associated two‐track model of ETBF carcinogenesis. The table summarizes the major mechanistic questions, a concise experimental strategy, and the result that would support the model. Evidence status indicates whether each row arises primarily from a falsifiable hypothesis [H] or from evidence‐based synthesis extended into a testable prediction [S] → [H]. ALDH, aldehyde dehydrogenase; CMS4, consensus molecular subtype 4; CRC, colorectal cancer; CSC, cancer stem cell; ETBF, enterotoxigenic Bacteroides fragilis; KO, knockout; NOD1, nucleotide‐binding oligomerization domain‐containing protein 1; OMV, outer membrane vesicle.

Hypothesis	Key experiment	Supportive result	Evidence status
Claudin‐4 expression level is the primary determinant of Track 1 cell‐selective BFT surface activity	Established by White et al., 2026 genome‐wide CRISPR‐KO screen in HT29 colonic epithelial cells; validated in polarized T84 monolayers	~20‐fold resistance in C4‐KO; ~500‐fold in C4/C3‐DKO; full sensitivity restored by GFP‐claudin‐4 genetic rescue proportional to expression level	[E]
OMV endocytic competence governs Track 2 (NOD1/stemness) cell‐selective response, independently of claudin‐4 expression	Block dynamin‐dependent endocytosis and macropinocytosis in claudin‐4‐competent cells; measure Track 1 E‐cadherin cleavage and Track 2 NOD1/CSC output separately	Track 2 NOD1/NOTCH1 signaling selectively abrogated; Track 1 E‐cadherin cleavage unaffected by endocytic blockade	[H]
BFT isoforms show track bias	Compare BFT‐1, BFT‐2, and BFT‐3 for NOD1 binding and E‐cadherin cleavage kinetics	Stronger NOD1 binding for BFT‐1; greater E‐cadherin cleavage for BFT‐2	[H]
OMV trafficking preferentially enables intracellular signaling	Compare free versus OMV‐associated BFT and block OMV uptake/endosomal acidification	NOD1/NOTCH1‐associated signaling depends on OMV trafficking, whereas early surface E‐cadherin cleavage is less affected	[S] → [H]
ETBF contributes to mesenchymal and stemness‐associated tumor programs	Test ETBF exposure in CRC organoid/tumor models for CMS4‐like, NOD1/NOTCH1, and ALDH‐associated features	Enrichment of mesenchymal and stemness‐related signatures after ETBF exposure	[H]
Extra‐colonic ETBF effects are detectable in diffuse‐type settings	Assess ETBF/BFT‐associated signatures in CDH1‐wild‐type mammary or gastric models and in diffuse‐type human tumors	Reduced membrane E‐cadherin, increased discohesion, and enrichment of Track 2 biomarkers	[S] → [H]
*bft* allele composition and sampling compartment explain part of reported ETBF–CRC heterogeneity	Retrospective isoform‐resolved genotyping of banked ETBF isolate collections for *bft* allele, stratified by sampling compartment (stool, mucosal biopsy, tumor) and by clinical endpoint (gene detection, toxin protein, serology)	Association strength varies systematically with *bft* allele and is greater in mucosa‐ and tumor‐derived isolates than in stool‐derived isolates; serological endpoints show stronger association than cross‐sectional PCR detection	[S] → [H]

## 7 Conclusions

ETBF is no longer merely a microbial correlate of colorectal cancer; it is a mechanistically intelligible, molecularly druggable oncobiotic driver. Prior linear models of BFT biology, focusing exclusively on extracellular junctional disruption, failed to account for the pathogen's capacity to induce profound chemoresistance, systemic metastatic priming, and durable epigenetic memory.

The two‐track model addresses these anomalies by integrating isoform‐specific structural bias, a candidate route of intracellular topological access, and feed‐forward inflammatory‐to‐stemness convergence. Its components carry unequal evidential weight, and we have tried to mark that inequality rather than smooth it: the surface program is established, the epigenetic memory arm has independent colonic support, and the NOD1‐dependent intracellular arm remains a hypothesis awaiting demonstration in colonic epithelium. The framework's value lies less in what it asserts than in the specificity of what it predicts. This framework transforms our understanding of ETBF from a generic inflammatory trigger into a sophisticated modulator of the tumor microenvironment that actively drives the aggressive CMS4 phenotype. Ultimately, translating this mechanistic clarity into clinical practice—guided by isoform‐resolved diagnostics, precision phage eradication, and track‐specific epigenetic interventions—represents a highly tractable and necessary frontier in precision oncobiome medicine.

## Conflict of interest

The authors declare no conflict of interest.

## Author contributions

KC, S‐HY, and K‐JR conceived and designed the project and carried out the investigation. KC and K‐JR prepared the figures. KC, S‐HY, and K‐JR wrote the original draft, and all authors reviewed and edited the manuscript. KC and K‐JR supervised the project and were responsible for project administration.

## Data Availability

Data sharing is not applicable to this article, as no new data were created or analyzed in this study.
